# Structure—Property—Performance Relationships in Thermoplastic Polyurethane: Influence of Infill Density and Surface Texture

**DOI:** 10.3390/polym17192716

**Published:** 2025-10-09

**Authors:** Patricia Isabela Brăileanu, Marius-Teodor Mocanu, Tiberiu Gabriel Dobrescu, Dan Dobrotă, Nicoleta Elisabeta Pascu

**Affiliations:** 1 Department of Robotics and Manufacturing Systems, Faculty of Industrial Engineering and Robotics, National University of Science and Technology POLITEHNICA Bucharest, 060042 Bucharest, Romania; patricia.braileanu@upb.ro (P.I.B.); tiberiu.dobrescu@upb.ro (T.G.D.); nicoleta.pascu@upb.ro (N.E.P.); 2 National Institute for Laser, Plasma and Radiation Physics Romania, 077125 Măgurele, Romania; 3 Doctoral School of Industrial Engineering and Robotics, Faculty of Industrial Engineering and Robotics, National University of Science and Technology POLITEHNICA Bucharest, 060042 Bucharest, Romania; 4 Faculty of Engineering, Lucian Blaga University of Sibiu, 550024 Sibiu, Romania

**Keywords:** additive manufacturing (AM), fused deposition modelling (FDM), fused filament fabrication (FFF), thermoplastic elastomers, internal structure, surface morphology, ball-on-disc, wear

## Abstract

This study investigates the structure–property–performance (SPP) relationships of two thermoplastic polyurethanes (TPUs), FILAFLEX FOAMY 70A and SMARTFIL^®^ FLEX 98A, manufactured by fused filament fabrication (FFF). Disc specimens were produced with varying gyroid infill densities (10–100%) and Archimedean surface textures, and their tribological and surface characteristics were analyzed through Ball-on-Disc tests, profilometry, and optical microscopy. SMARTFIL^®^ FLEX 98A exhibited a sharp reduction in the coefficient of friction (μ) with increasing infill, from 1.174 at 10% to 0.371 at 100%, linked to improved structural stability at higher densities. In contrast, FILAFLEX FOAMY 70A maintained a stable but generally higher coefficient of friction (0.585–0.729) across densities, reflecting its foamed microstructure and bulk yielding behavior. Surface analysis revealed significantly higher roughness in SMARTFIL^®^ FLEX 98A, while FILAFLEX FOAMY 70A showed consistent roughness across infill levels. Both TPUs resisted inducing abrasive wear on the steel counterpart, but their stress-accommodation mechanisms diverged. These findings highlight distinct application profiles: SMARTFIL^®^ FLEX 98A for energy-absorbing, deformable components, and FILAFLEX FOAMY 70A for applications requiring stable surface finish and low adhesive wear. The results advance the design of functionally graded TPU materials through the controlled tuning of infill and surface features.

## 1. Introduction

Additive manufacturing (AM) defines the family of processes based on layer-by-layer fabrication directly from digital models [1], enabling geometries that are otherwise difficult or impossible to achieve, as well as functional integration. The origins of AM can be traced back to the 1980s, when the first stereolithography technologies were developed (Hull, 1986) [2]. Since then, AM has expanded to a wide range of materials and processes, with several comprehensive overviews available in literature such as Gibson et. al. [3] and Badiru et. al. [4]. In this study, the focus is on Material Extrusion (MEX) applied to thermoplastic polyurethane (TPU), where the internal architecture and surface external pattern are expected to significantly influence tribological performance.

Among the diverse AM technologies, Fused Filament Fabrication (FFF), also referred to as MEX in M. Batista et al. study, stands out due to its cost-efficiency, operational simplicity and integral material versatility, making it widely adopted for producing polymeric and resin-based components [1,5,6]. Prior studies, including those of A. P. Golhin et al. highlight the importance of polymers as the basis of 3D printing, prized for their affordability, widespread availability, ease of processing and diverse aesthetic options [7].

Thermoplastic Polyurethane (TPU) is particularly notable within the realm of flexible polymers. A versatile thermoplastic elastomer, as reported by V. K. Yadavalli et al., TPU boasts a unique combination of properties, including exceptional flexibility, high elongation, superior abrasion resistance and excellent resistance to oils and greases, contributing to its escalating popularity across numerous industries [8]. Yadavalli observed a distinct elastomeric behavior in TPU that originates from its macromolecular architecture, which consists of mobile aliphatic polyether segments (soft segments, SS) and rigid aromatic groups (hard segments, HS) that naturally self-organize into discrete soft and hard domains [8]. This built-in biphasic nature allows for a broad spectrum of customizable characteristics, by modulating the ratio of SS to HS and the structural morphology. As a result, TPU’s properties can be precisely tuned from a very soft, rubber-like feel to a more rigid, plastic-like consistency. This tunability results in outstanding performance attributes, such as excellent wear resistance, high tensile strength and superior chemical resistance [8]. Its biocompatibility and hemocompatibility further position TPU as an optimal material for biomedical applications, including the fabrication of customized orthotics [8,9]. Advanced foaming technologies investigated by M. C. Iacob et al., exemplified by materials like colorFabb^®^ VarioShore TPU, expand this customizations capability, allowing for the manipulation of 3D print properties by adjusting process parameters such as printing temperature, speed, or flow ratio to control the level of expansion and, consequently, the material’s stiffness [10]. Specifically, Iacob et al. highlight that TPU’s hardness can be adjusted between Shore 55A (fully foamed) and Shore 92A (unfoamed) by varying the nozzle temperature within a range of 190–250 °C, enabling volumetric expansion by approximately 1.4–1.6 times its original volume within a single print [10].

The capacity of AM processes to generate complex internal geometries and external surface characteristics is fundamental to modulate the structure–property–performance (SPP) relationships of materials. This architectural control facilitates the functional customization of components, thereby enhancing their mechanical, tribological and thermal attributes to meet precise application demands. A critical parameter, identified by F. He et al. and M. N. M. Norani et al., in achieving this customization is the infill density and its corresponding infill pattern. Infill density directly dictates the amount of material within a 3D printed part, significantly impacting its mechanical strength, overall weight and printing duration [11,12]. Generally, M. N. M. Norani observed that infill densities correlate with increased strength and viscosity, a result of improved adhesive bonding between polymer molecules [11].

The selection of specific infill patterns, such as gyroid, honeycomb, zigzag or grid, exerts a profound influence on the mechanical behavior of 3D prints [8,10,13]. M. N. M. Norani highlights that controlling internal lattice structures through infill parameters can augment compressive strength and energy absorption [11]. These capabilities are particularly instrumental in producing functionally graded materials (FGMs), where properties like stiffness can be systematically varied within a single component by adjusting infill density and patterns via G-code modifications [10,14]. This advanced approach finds practical utility in developing personalized foot orthoses (insoles) with adjustable stiffness profiles tailored to plantar pressure measurements [9,10], and designing wheelchair cushions that offer improved pressure offloading and prevention of bottoming-out, as shown by R. Tilley et al.’s study [14].

Beyond internal architecture, the surface texture of FDM^®^ printed parts substantially affects their functional behavior, especially in tribological contexts concerning friction, wear and lubrication [1]. Due to their inherent layer-by-layer deposition, AM parts, particularly those fabricated via FDM^®^, typically exhibit anisotropy, stair-step surface roughness and internal porosity [1,7]. These surface irregularities often compromise wear resistance and frictional stability, as interlayer discontinuities act as stress concentrators and sites for wear initiation, as highlighted by R. Subramani et al. [1].

While generally, smoother surfaces contribute to reduced friction and wear rates, strategically designed surface textures can enhance tribological outcomes by facilitating lubrication and effectively trapping wear debris [15,16]. Essential printing parameters such as layer thickness, build orientation, printing speed, nozzle diameter and temperature are decisive factors in determining surface roughness [7,17,18,19,20]. Inconsistent strand welding conditions, influenced by material flow, strand width, deposition temperature and printing speed, can lead to non-uniform internal stresses, surface distortions and diminished bonding, all of which adversely impact surface texture [21,22]. To mitigate these inherent limitations and enhance surface durability, various surface engineering and post-processing techniques are employed, including mechanical sanding, vapor smoothing, thermal annealing and laser polishing [1,23,24,25]. While the literature has extensively reported on the mechanical properties of FDM^®^-printed TPU, tribological behavior remains underexplored, particularly in relation to the synergistic influence of infill density and surface texture. Most existing studies focus either on internal architecture [21,22] or on post-processing methods [23,24,25], without providing an integrated evaluation of tribological performance under standardized conditions. This work addresses this gap by systematically comparing foamed and non-foamed TPU filaments across a wide range of infill densities, thereby establishing structure–property–performance relationships that have not been previously documented.

However, the complex interplay between texture parameters (e.g., shape, size, distribution density) and surface functionality poses significant design challenges, increasingly mandating the integration of advanced computational tools like machine learning algorithms for predictive optimization, as highlighted by K. Chen et al. [26].

Even with substantial developments in polymer AM, several unresolved issues hinder a complete understanding and optimization of the structure–property–performance relationships in TPU components [10,27,28,29,30]. Current research on the tribological properties of 3D printed polymers is comparatively limited (only 3% of studies, versus 12% focusing on mechanical properties, as reported by N. Stoimenov et al.) [31], highlighting a pressing need for a generalized model that integrates the influence of diverse materials and printing parameters on tribological properties under various conditions [12]. Practical challenges associated with 3D printing extremely flexible TPU filaments (e.g., 60A and 70A Shore hardness), including issues such as nozzle clogs, filament buckling and compromised print quality, also represent significant informational gaps in current literature [9].

This study aims to address these critical limitations by systematically investigating the complex structure–property–performance relationships in thermoplastic polyurethane, with a distinct focus on the synergistic influence of infill density and surface texture of two type of TPU: FILAFLEX FOAMY 70A (Recreus Industries S.L., Alicante, Spain) and SMARTFIL^®^ FLEX 98A (INNOVATEFIL^®^, Smart Materials 3D, Alcalá la Real, Jaén, Spain). By examining how different gyroid infill densities influence both tribological behavior and surface texture of 3D printed TPU specimens, this research aims to generate comprehensive data and actionable insights that address gaps in the existing literature. The novelty of this work resides in its integrated approach to characterizing the combined effects of internal architecture and external surface features on TPU’s performance, thereby advancing the design and manufacturing of customized, high-performance polymeric components for diverse applications where variable stiffness and controlled friction are important. This TPU material-specific investigation will contribute to the development of sturdier predictive models and the optimization of printing strategies for flexible polymeric materials, facilitating their broader industrial adoption.

Therefore, despite increasing research interest in additive manufacturing of flexible polymers, the combined effect of infill density and surface texture on the tribological behavior of TPUs remains insufficiently explored. This study addresses this gap by systematically investigating the synergistic influence of gyroid infill density and Archimedean surface patterns on the tribological and surface properties of two distinct TPUs. The novelty of this work lies in its integrated approach to structure–property–performance relationships, providing insights that support the design of customized TPU components with tailored frictional and mechanical responses.

## 2. Materials and Methods

The investigation focuses on elucidating SPP relationships, with particular attention to factors such as infill density and surface texture, as modulated by processing parameters through the FDM^®^ method. Two distinct types of thermoplastic polyurethane filaments were investigated: SMARTFIL^®^ FLEX 98A and FILAFLEX FOAMY 70A.

SMARTFIL^®^ FLEX 98A, supplied by INNOVATEFIL^®^ (Smart Materials 3D, Alcalá la Real, Jaén, Spain), is a thermoplastic polyurethane specifically engineered with additives to facilitate the 3D printing of flexible objects exhibiting elasticity [32]. Distinguished by a higher hardness compared to other conventional flexible filaments, it offers improved printability across a broad spectrum of 3D printers, including both direct extrusion and Bowden systems [32]. This material is particularly suited for applications requiring robust performance under significant stress or vibration due to its excellent inter-layer adhesion and high impact resistance.

FILAFLEX FOAMY 70A, manufactured by Recreus Industries S.L. (Elda, Alicante, Spain), is a thermoplastic polyurethane filament that incorporates a dynamic foaming technology, which integrates a foaming agent uniformly distributed within the filament [33]. This agent activates at elevated temperatures during printing, imparting a foamy texture and appearance to the printed parts, which results in reduced weight and density. Table 1 summarizes the physical and mechanical properties of the thermoplastic polyurethane filaments used in this study, SMARTFIL^®^ FLEX 98A and FILAFLEX FOAMY 70A, as reported in their manufacturer data sheets [32,33].

The fabrication of all specimens commenced with the digital design of disc-shaped cylinders, measuring 40 mm in diameter and 10 mm in height, using computer-aided design (CAD) software and subsequent export into stereolithography (STL) format. These 3D models were then imported into Creality Print 6.0 slicing software (Creality Co., Ltd., Shenzhen, China) for G-code generation. Fabrication was performed on a Creality K1C FDM^®^ printer (Creality Co., Ltd., Shenzhen, China), equipped with an enclosed build chamber, a 1.75 mm hardened steel nozzle and an all-metal direct-drive extruder. Given the hygroscopic nature of polymer filaments, all materials underwent pre-drying using a Creality Space Pi Filament Dryer (Creality Co., Ltd., Shenzhen, China) to prevent porosity and ensure data validity. Important FDM^®^ printing parameters, including nozzle temperature, build plate temperature and extrusion printing speeds, were individually optimized for each TPU material to achieve consistent interlayer adhesion and dimensional accuracy, as shown in Table 2.

The applied normal load of 5 N and sliding speed of 150 mm/s were selected to comply with ASTM G99-17 while simulating typical contact pressures encountered in polymeric components under moderate tribological stress. A nozzle temperature of 230 °C was employed for both TPUs, in accordance with manufacturer recommendations, to ensure consistent interlayer bonding and minimize void formation. Three replicates were produced for each condition to ensure statistical validity, and data were further analyzed by one-way ANOVA to evaluate the significance of observed differences (*p* < 0.05).

**Figure 1:**
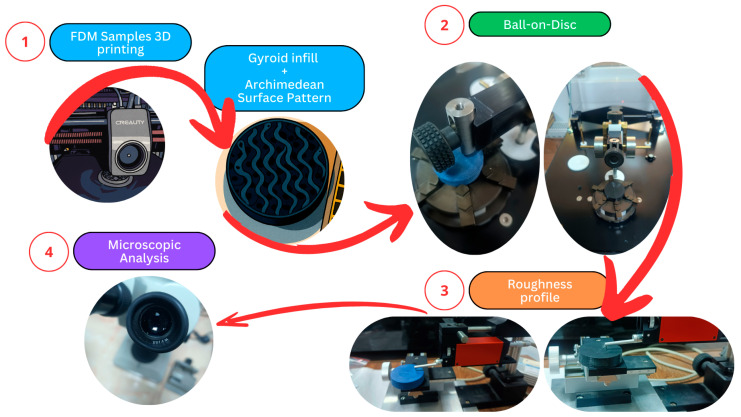
Overview of the experimental procedure for tribological, surface analysis and mechanical characterization of thermoplastic polyurethane (TPU) samples.

General slicing parameters included two perimeter walls, four solid top and bottom layers, an Archimedean pattern for these external surfaces, a gyroid sparse infill structure, and a standard 0.2 mm layer height. To assess the influence of internal architecture, specimens were printed with six distinct sparse infill densities: 10%, 30%, 50%, 70%, 90%, and 100% (in this case all layers adopted an Archimedean pattern, resulting in a fully solid component). To ensure statistical robustness, three specimens were produced for each material and infill density configuration. Following fabrication, specimens underwent post-processing, involving cleaning with compressed air to remove any impurities and wiping with 99% isopropyl alcohol (Kynita S.R.L., Budești, Vâlcea, Romania) to eliminate surface residues. Throughout fabrication and all testing measurements conducted on the TPU specimens, environmental conditions were strictly controlled at 24 ± 1 °C and approximately 50% relative humidity (RH). The experimental methodology is illustrated in Figure 1, which outlines the main stages of sample preparation and characterization.

Tribological performance was assessed via Ball-on-Disc experiments using a TRIBOtester system (Tribotechnic US LLC, San Francisco, CA, USA), adhering to the ASTM G99-17 standard [34]. In this configuration, the FFF printed TPU specimens served as the rotating disc, while a stationary spherical pin of Steel 100Cr6 (Tribotechnic US LLC, San Francisco, CA, USA), complying with DIN 17,230 standard [35], acted as the counter body. This steel ball had a nominal diameter of 6 mm, a Young’s modulus of 205 GPa, and a typical Poisson’s ratio of 0.33. All tests were conducted under ambient, dry sliding contact conditions, without external lubrication. The tests were performed with a normal load of 5 N, a sliding speed of 150 mm/s, and a total sliding length of 300 m, resulting in a calculated Hertz pressure of 231 MPa. The steel ball was mounted perpendicularly to the specimen’s rotating surface to ensure consistent contact.

For quantitative assess surface modification, profilometric analysis was systematically conducted on all worn specimens using a stylus profilometer (Tribotechnic US LLC, San Francisco, CA, USA). Multiple linear profilometric measurements were conducted, with three independent measurements per specimen averaged to enhance data reliability.

For each infill density and TPU type, three specimens were fabricated to ensure statistical robustness. Mean values and standard deviations were calculated from these replicates. Surface roughness was quantified using a stylus profilometer (accuracy ± 0.1 µm), while tribological tests were carried out on a TRIBOtester system with load precision ± 0.05 N, in compliance with ASTM G99-17.

As a final step, complementary microscopic analysis of worn surfaces was performed using a digital stereo optical microscope (NOVEX RZT-SF, Euromex Microscopen B. V., Duiven, The Netherlands), equipped with a CMEX DC.1300× USB camera (Euromex Microscopen B. V., Duiven, The Netherlands) and ImageFocus v 2.5 software (Euromex Microscopen B. V., Duiven, The Netherlands). Specimens were secured in an adjustable holder, and images were captured at a total magnification of 20× (10× objective with 2× magnification base). This facilitated the evaluation of surface appearance before and after tribological testing, specifically for observing if wear tracks and associated damage appear.

## 3. Results

### 3.1. Tribological Performance of TPU

The tribological characteristics of FILAFLEX FOAMY 70A manufactured with gyroid infill structures were evaluated across infill densities of 10–100% and the results are summarized in Table 3.

Similarly, SMARTFIL^®^ FLEX 98A specimens were analyzed under identical tribological conditions across infill densities from 10% to 100%, with results shown in Table 4.

For the 10% FILAFLEX FOAMY 70A gyroid infill sample, the friction coefficient (*μ*) started at 0.734, with an average value of 0.585, a minimum of 0.483 and a maximum of 0.902. Visual inspection post-test revealed no detectable profile on the polymer sample and no mark on the steel ball, indicating minimal macroscopic wear on both surfaces.

**Figure 2:**
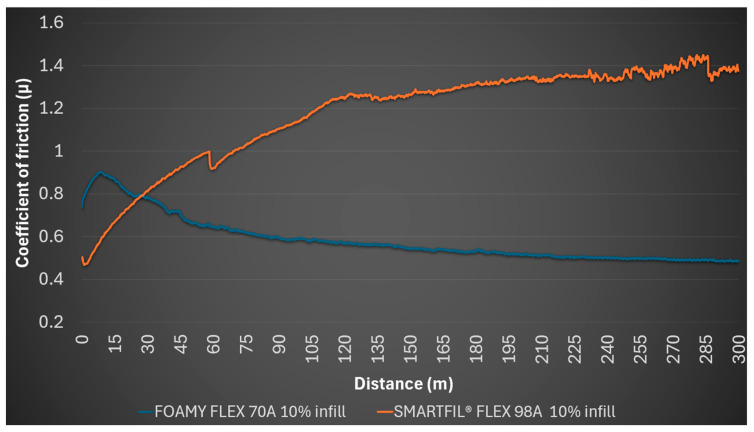
Evolution of coefficient of friction (*μ*) over sliding distance for 10% infill TPU samples.

Instead, for the 10% SMARTFIL^®^ FLEX 98A gyroid infill sample, the coefficient of friction at the test start was 0.495, reaching a high average of 1.174, with a minimum of 0.467 and a maximum of 1.45. A distinctive observation for this sample was the deformation of internal structures due to the pressure exerted by the steel ball, indicating a lack of structural integrity under tribological loading at this low infill density. No detectable profile was formed on the sample, and no mark was left on the steel ball. Figure 2 shows that SMARTFIL^®^ FLEX 98A exhibits continuously increasing coefficient of friction, while FOAMY FLEX 70A maintains lower and more stable values.

At 30% FILAFLEX FOAMY 70A infill sample, the starting coefficient of friction was 0.647, maintaining this value as the average coefficient of friction, with a minimum of 0.612 and a maximum of 0.840. In line with the 10% infill, no detectable profile or mark on the steel ball was observed.

**Figure 3:**
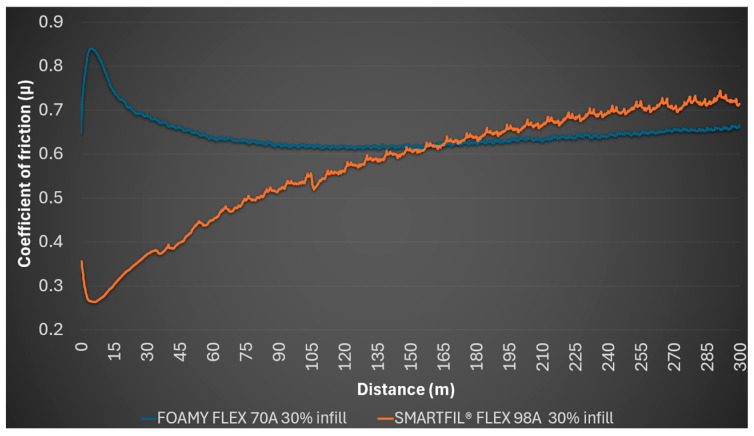
Evolution of coefficient of friction (*μ*) over sliding distance for 30% infill TPU samples.

The 30% SMARTFIL^®^ FLEX 98A infill sample began with a coefficient of friction of 0.356, had an average of 0.572, a minimum of 0.264 and a maximum of 0.744. Continuous data showed the coefficient of friction fluctuated and generally increased from an initial 0.334 to around 0.695 at 222.15 m. No detectable wear profile was formed, and the steel ball remained unmarked. Figure 3 illustrates the coefficient of friction variation over the sliding distance for both materials at 30% infill density.

The 50% FILAFLEX FOAMY 70A gyroid infill sample exhibited a starting coefficient of friction of 0.784, an average of 0.647, a minimum of 0.585 and a maximum of 0.784. Consistent low wear was noted, with no detectable profile on the sample or mark on the steel ball.

**Figure 4:**
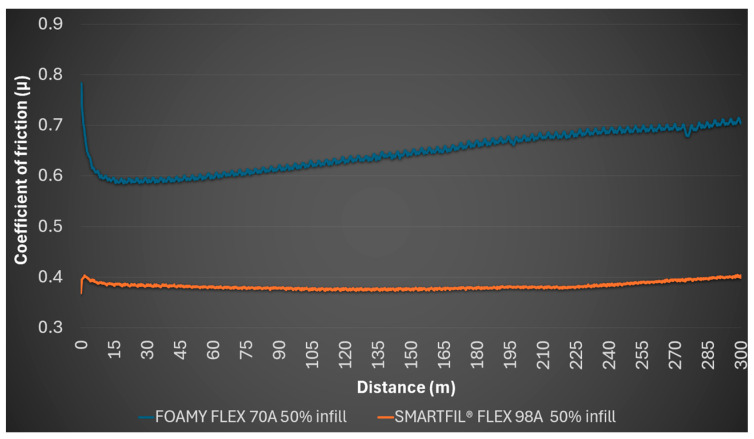
Evolution of coefficient of friction (*μ*) over sliding distance for 50% infill TPU samples.

For the 50% SMARTFIL^®^ FLEX 98A gyroid infill material, it started at a lower coefficient of friction of 0.368, but rapidly increased to an average of 0.382, with a minimum of 0.368 and a maximum of 0.404. The Archimedean pattern surfaces showed no detectable profile or mark on the steel ball. The coefficient of friction trends for FILAFLEX FOAMY 70A and SMARTFIL^®^ FLEX 98A at 50% infill are illustrated in Figure 4.

The 70% density FILAFLEX FOAMY 70A sample measured an initial coefficient of friction of 0.480, rising to an average of 0.729, with a minimum of 0.480 and a maximum of 0.790. Post-test observations showed no detectable profile on the sample and no mark on the steel ball.

**Figure 5:**
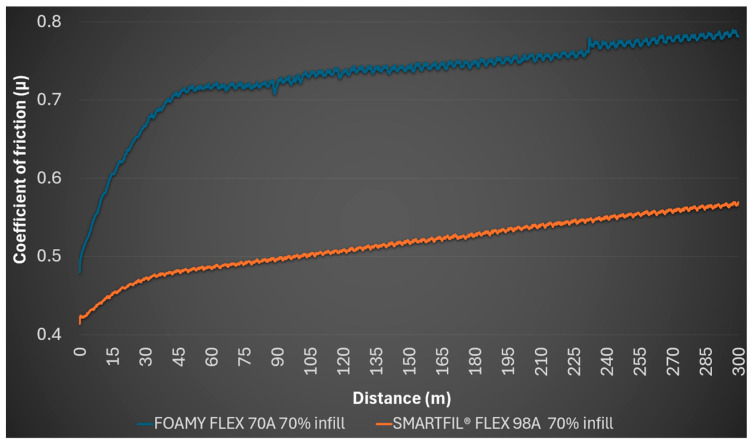
Evolution of coefficient of friction (*μ*) over sliding distance for 70% infill TPU samples.

The 70% SMARTFIL^®^ FLEX 98A infill sample showed a starting coefficient of friction of 0.414, an average of 0.515, a minimum of 0.414, and a maximum of 0.569. No detectable profile was observed, and the steel ball remained unmarked. Figure 5 presents the friction coefficient evolution for both FILAFLEX FOAMY 70A and SMARTFIL^®^ FLEX 98A samples at 70% infill.

The 90% FILAFLEX FOAMY 70A samples showed a starting coefficient of friction of 0.482, an average of 0.717, a minimum of 0.482 and a maximum of 0.764. The wear behavior remained consistent, with no detectable profile or mark on the steel ball. The wear behavior was consistent, showing merely a faint abrasion trace on the sample and slight debris on the steel ball, without any discernible profile or marking on the ball itself.

**Figure 6:**
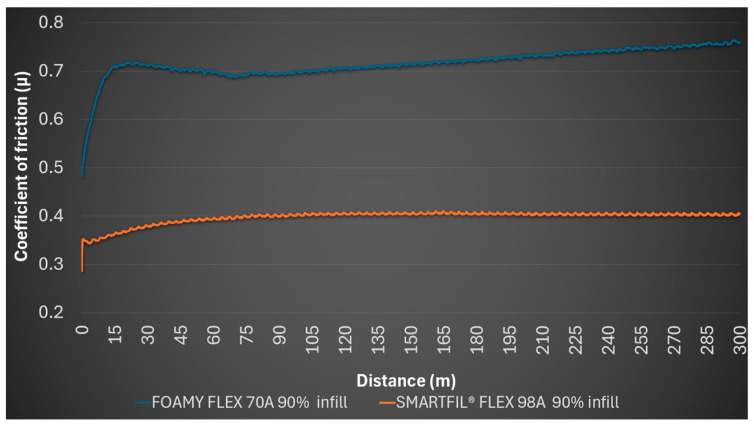
Evolution of coefficient of friction (*μ*) over sliding distance for 90% infill TPU samples.

For the 90% SMARTFIL^®^ FLEX 98A gyroid infill material with Archimedean surface pattern, the initial coefficient of friction was 0.286, with an average of 0.398, a minimum of 0.286 and a maximum of 0.41. No detectable profile on the sample or mark on the steel ball was reported. Figure 6 illustrates the coefficient of friction variation for FILAFLEX FOAMY 70A and SMARTFIL^®^ FLEX 98A at 90% infill density over the testing distance.

**Figure 7:**
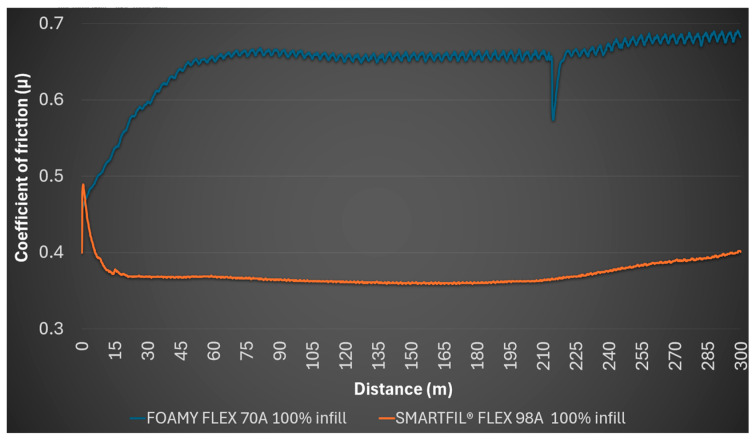
Evolution of coefficient of friction (*μ*) over sliding distance for 100% infill TPU samples.

Finally, the FILAFLEX FOAMY 70A 100% gyroid infill sample began with a coefficient of friction of 0.445, achieving an average of 0.647, with a minimum of 0.445 and a maximum of 0.691. Across the test duration, the coefficient of friction generally increased from approximately 0.421 at the start to about 0.666, with a notable drop to approximately 0.574 at around 214.8 m of sliding distance. Despite this fluctuation, no detectable profile on the sample or mark on the steel ball was observed. Overall, FILAFLEX FOAMY 70A samples consistently demonstrated low wear on the steel counterpart across all tested infill densities. The average coefficient of friction showed variability, peaking at 70% infill density (0.729), suggesting a complex relationship between infill density and friction in this foamy TPU material. Figure 7 displays the evolution of the coefficient of friction for both TPUs at 100% infill density across the sliding distance.

Also, the SMARTFIL^®^ FLEX 98A 100% gyroid infill sample had a starting coefficient of friction of 0.4, an average of 0.371, a minimum of 0.359, and a maximum of 0.489. The coefficient of friction initially increased, then stabilized and slightly decreased, fluctuating between 0.36 and 0.37 in most of the tests. Minor fine scratches appeared on both the specimen and the steel ball, yet no detectable profile was present.

### 3.2. Comparative Tribological Analysis

A comparative analysis between FILAFLEX FOAMY 70A and SMARTFIL^®^ FLEX 98A reveals distinct tribological responses influenced by infill density and surface texture.

Coefficient of friction trends:

- SMARTFIL^®^ FLEX 98A exhibited a significantly higher average coefficient of friction at 10% infill density (1.174) compared to all other samples, including FILAFLEX FOAMY 70A at the same density (0.585). This extreme value for FLEX 98A at 10% infill was accompanied by observations of deformed internal structures. This suggests that at very low infill densities, the material’s structural integrity is compromised, leading to substantial deformation under the applied Hertzian pressure of 231 MPa, which in turn results in higher energy dissipation and friction.
- As the infill density of SMARTFIL^®^ FLEX 98A increased from 10% to 50%, the average coefficient of friction generally decreased considerably (from 1.174 at 10% to 0.382 at 50%). This trend indicates that higher infill densities provide enhanced structural support, reducing bulk material deformation and consequently lowering friction. At 90% and 100% infill, the average coefficient of friction remained low (0.398 and 0.371 respectively).
- In contrast, FILAFLEX FOAMY 70A did not show such a pronounced decrease in coefficient of friction with increasing infill density. Its average coefficient of friction values generally remained within a range of 0.585 to 0.729, which were typically higher than most infill SMARTFIL^®^ FLEX 98A samples (e.g., Flex 98A 50%). The highest average coefficient of friction for FILAFLEX FOAMY 70A was observed at 70% infill (0.729). This behavior might be attributed to its inherent foamy nature, which could lead to greater surface compliance and adhesion, influencing friction differently from the denser SMARTFIL^®^ FLEX 98A at similar infill levels.

Wear behavior and surface performance:

- A consistent finding for both TPU materials, across almost all infill densities and surface conditions, was the observation of no detectable wear profile on the sample with no particular mark on the steel ball. This indicates that under the tested conditions (5 N normal load, 231 MPa Hertz pressure), neither TPU induced significant abrasive wear on the harder steel ball. The materials primarily deformed elastically or plastically without substantial material removal that would leave a visible wear track or transfer material to the steel sphere.
- The notable exception was SMARTFIL^®^ FLEX 98A at 10% infill, where the material’s internal structures appeared to deform significantly under the applied pressure. This suggests that very low infill density in this specific TPU results in a drastic reduction in load-bearing capacity, leading to bulk deformation rather than a superficial tribological response.

**Figure 8:**
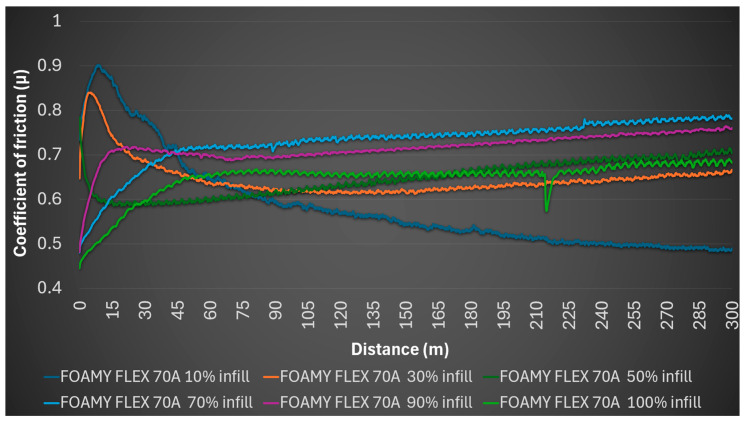
Effect of infill density on the coefficient of friction (*μ*) of FILAFLEX FOAMY 70A during dry sliding.

Therefore, the infill density profoundly influences the tribological performance of these TPU materials, particularly for SMARTFIL^®^ FLEX 98A where lower densities led to structural deformation and exceptionally high friction. FILAFLEX FOAMY 70A, possibly due to its foamy characteristic, exhibited a more consistent, albeit generally higher, coefficient of friction range. Both materials generally showed excellent resistance to inducing wear on the steel counterpart. Figure 8 illustrates the coefficient of friction variation for FILAFLEX FOAMY 70A samples fabricated with different infill densities (10% to 100%) under dry sliding conditions.

**Figure 9:**
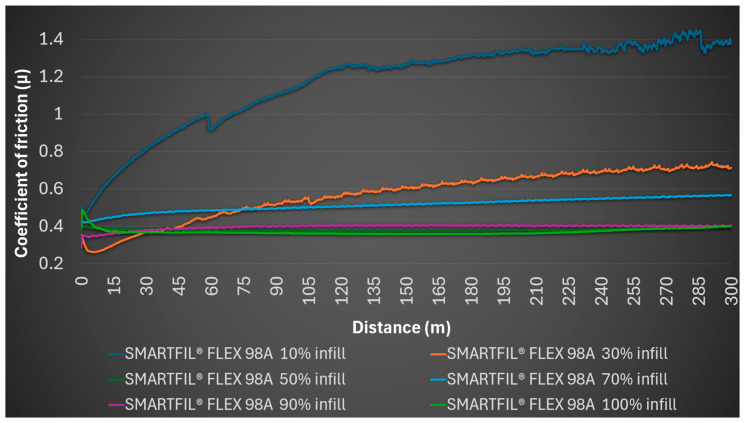
Effect of infill density on the coefficient of friction (*μ*) of SMARTFIL^®^ FLEX 98A during dry sliding.

Figure 9 shows the evolution of the coefficient of friction for SMARTFIL^®^ FLEX 98A with varying infill densities, complementing the results presented previously for FILAFLEX FOAMY 70A.

Figure 8 and Figure 9 demonstrate a clear divergence in material response: FLEX 98A exhibits statistically significant improvements in friction coefficient reduction with increasing infill (*p* < 0.01), while FOAMY 70A remains within a narrow range (*p* > 0.05). These differences highlight distinct load-bearing and deformation mechanisms inherent to the two TPUs.

### 3.3. Surface Roughness and Tribological Response of TPU

The correlation between surface roughness and frictional response was evident: higher R_a_ and R_q_ values in FLEX 98A corresponded to lower coefficient of friction at higher infill, indicating that increased structural rigidity compensates for rougher surface topography. In contrast, FOAMY 70A exhibited relatively stable roughness values, consistent with its foamed morphology, which resulted in more uniform coefficient of friction values across densities.

Surface characterization, roughness parameters (ISO 4287 amplitude and material ratio [36]) and observations on material response to applied load were analyzed to establish SPP relationships.

The surface analysis of FILAFLEX FOAMY 70A samples, manufactured with an Archimedean top and bottom pattern, revealed distinct topographical features and responses under load, varying with gyroid infill density. It was generally noted that for many samples, a clear profile was not discernible, and no trace was left on the steel ball used in testing, suggesting either significant elastic recovery or minimal material transfer. However, numerical roughness parameters were consistently obtained. The average amplitude parameters and roughness profiles of FILAFLEX FOAMY 70A specimens manufactured with various gyroid infill densities are summarized in Table 5.

Surface roughness of FILAFLEX FOAMY 70A:

- Average arithmetic mean deviation (*R_a_*) ranged from 1.89 µm (30% infill density) to 4.09 µm (70% infill). The root mean square roughness (*R_q_*) followed a similar trend, varying from 2.71 µm (30% gyroid infill density) to 5.18 µm (70% infill).
- The maximum height of profile (*R_z_*) averaged between 13.79 µm (30% infill) and 21.13 µm (70% infill), while the total height of profile (*R_t_*) spanned from 19.3 µm (30% infill) to 27.26 µm (70% gyroid infill).
- Maximum peak height (*R_p_*) varied from 4.07 µm (30% infill) to 6.47 µm (90% infill), and maximum valley depth (*R_v_*) from 9.72 µm (30% infill) to 14.73 µm (70% infill density). The mean height of profile elements (*R_c_*) was observed between 6.9 µm (30% infill) and 13.9 µm (70% filling rate). These parameters indicate a relatively consistent roughness magnitude across the tested infill densities, with 70% infill density exhibiting slightly higher average values for several parameters.

Surface morphology of FILAFLEX FOAMY 70A:

- All tested FILAFLEX FOAMY 70A samples exhibited a negative skewness (*R_sk_*), ranging from −1.02 (90% infill) to −1.45 (30% infill). This consistently indicates a surface dominated by valleys.
- Kurtosis (*R_ku_*) values varied from 3.53 (90% gyroid infill) to 5.85 (30% gyroid infill). Since *R_ku_* > 3, these surfaces are characterized by sharp peaks and valleys (spiky distribution).

Material ratio parameters FILAFLEX FOAMY 70A:

- The material ratio (*R_mr_*) at a cut level of 1 µm under the highest peak ranged from 1.33% (30% infill density) to 4.06% (70% infill).
- Profile section height difference (*R_dc_*), representing the effective depth of the functional surface profile, varied from 2.83 µm (30% infill) to 7.67 µm (70% infill).

The material ratio parameters of FILAFLEX FOAMY 70A specimens at various gyroid infill densities are summarized in Table 6.

SMARTFIL^®^ FLEX 98A samples, also with a gyroid infill pattern, demonstrated distinct surface characteristics and a marked deformation behavior under load. A consistent observation was that the area where the ball pressed seemed to have some deformation and the internal structures probably deformed from the pressure exerted by the ball, without leaving a trace on the steel ball. This suggests a primary mechanism of localized plastic deformation or structural collapse rather than material transfer to the counter surface. The average amplitude parameters and roughness profiles of SMARTFIL^®^ FLEX 98A specimens manufactured with various gyroid infill densities are summarized in Table 7.

Surface roughness of SMARTFIL^®^ FLEX 98A:

- Average arithmetic means deviation (*R_a_*) showed a broad range, increasing significantly with higher infill densities, from 2.34 µm (10% infill density) to 10.74 µm (70% infill). Similarly, *R_q_* ranged from 4.17 µm (10% infill) to 14.13 µm (70% infill).
- The maximum height of profile (*R_z_*) also increased with infill density, from 22.53 µm (10% infill density) to 59 µm (70% gyroid infill). The total height of the profile (*R_t_*) varied from 24.63 µm (10% infill) to 65.57 µm (70% infill).
- *R_p_* values increased from 3.01 µm (10% gyroid infill) to 12.89 µm (70% infill), and *R_v_* from 19.5 µm (10% infill density) to 46.1 µm (70% infill ratio). *R_c_* also showed an upward trend, from 17.03 µm (10% infill) to 45.4 µm (70% infill). These results clearly demonstrate that increasing infill density leads to a substantially rougher surface for SMARTFIL^®^ FLEX 98A.

Surface morphology of SMARTFIL^®^ FLEX 98A:

- All SMARTFIL^®^ FLEX 98A samples exhibited a negative skewness (*R_sk_*), ranging from −1.67 (90% infill) to −3.24 (10% infill), indicating a consistent valley-dominated surface. The magnitude of negative skewness was particularly high at lower infill densities (10%, 30% infill densities).
- Kurtosis (*R_ku_*) values varied widely, from 4.93 (90% infill) to 13.4 (10% infill gyroid density). With *R_ku_* > 3, these surfaces are generally characterized by sharp peaks and valleys, with the 10% infill showing an exceptionally spiky profile.

Material ratio parameters of SMARTFIL^®^ FLEX 98A:

- The material ratio (*R_mr_*) at a cut level of 1 µm under the highest peak ranged from 1.34% (70% and 90% infill density) to 6.47% (10% infill density).
- Profile section height difference (*R_dc_*) varied from 1.43 µm (10% infill) to 17.5 µm (70% infill).

The material ratio parameters of SMARTFIL^®^ FLEX 98A specimens at various gyroid infill densities are summarized in Table 8.

### 3.4. Comparative Analysis of Surface Roughness in TPU

A comparative analysis between FILAFLEX FOAMY 70A and SMARTFIL^®^ FLEX 98A reveals distinct differences in their surface textures and responses to mechanical loading, influenced by infill density.

Initial surface roughness:

- SMARTFIL^®^ FLEX 98A consistently presented significantly rougher surfaces than FILAFLEX FOAMY 70A across comparable infill densities (10%, 30%, 70%, 90% infill densities). For instance, at 70% infill, SMARTFIL^®^ FLEX 98A showed an average *R_a_* of 10.74 µm, whereas FILAFLEX FOAMY 70A exhibited an average *R_a_* of 4.09 µm. This trend is maintained by *R_q_*, *R_z_*, and *R_t_* parameters as well.
- For SMARTFIL^®^ FLEX 98A, surface roughness parameters (*R_a_*, *R_q_*, *R_z_*, *R_t_*, *R_p_*, *R_v_*, *R_c_*) increased substantially with increasing infill density, indicating that higher infill leads to a more pronounced topographical variation. In contrast, FILAFLEX FOAMY 70A maintained a relatively stable roughness magnitude across its tested infill densities.

**Figure 10:**
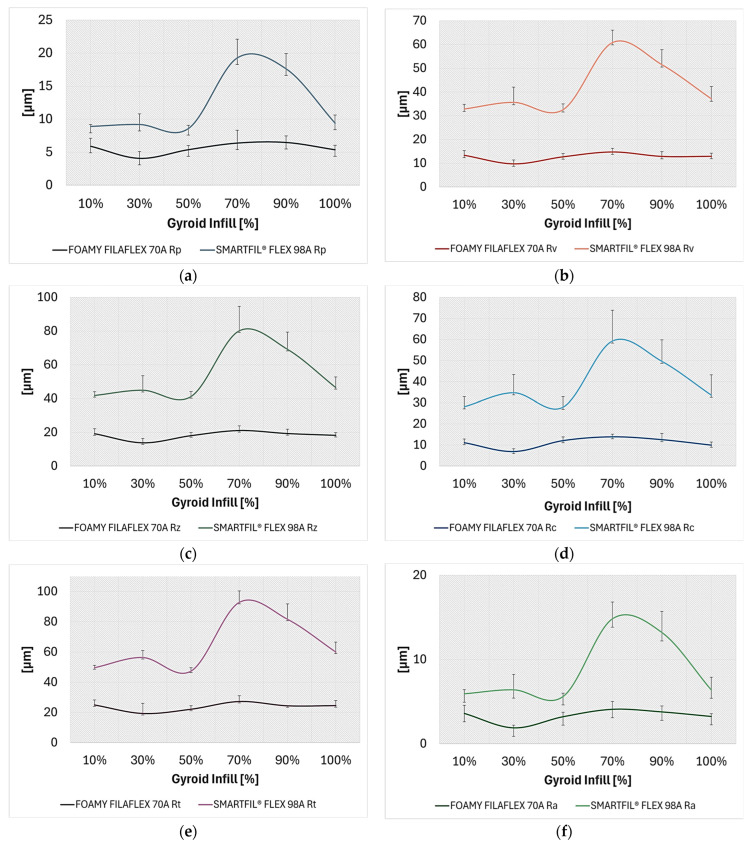
Evolution of the average amplitude parameters of TPU samples: (**a**) Average maximum profile peak height (*R_p_*); (**b**) Average maximum profile valley depth (*R_v_*); (**c**) Average maximum height of the profile (*R_z_*); (**d**) Average mean height of profile elements (*R_c_*); (**e**) Average total height of the profile (*R_t_*); (**f**) Average arithmetic means roughness (*R_a_*).

The evolution of surface roughness parameters for the TPU samples, in relation to different infill densities and materials, is illustrated in Figure 10a–f.

Surface morphology (skewness and kurtosis):

- TPU materials generally exhibited valley-dominated surfaces (negative *R_sk_*). However, SMARTFIL^®^ FLEX 98A, particularly at lower infill densities (10% infill: *R_sk_* −3.24), showed a more pronounced negative skewness compared to FILAFLEX FOAMY 70A (ranging from −1.02 to −1.45). This suggests deeper, more prominent valleys in SMARTFIL^®^ FLEX 98A, especially at lower infill.
- Regarding kurtosis, both materials presented spiky surfaces (*R_ku_* > 3), but SMARTFIL^®^ FLEX 98A displayed significantly higher *R_ku_* values, particularly at 10% infill density (13.4), indicating a much sharper distribution of peaks and valleys compared to FILAFLEX FOAMY 70A (ranging from 3.53 to 5.85).

Response to mechanical loading:

- A key distinction lies in the material response to contact. SMARTFIL^®^ FLEX 98A consistently exhibited deformation of internal structures under the ball’s pressure. This is evidenced by the substantial maximum wear track depths and areas measured for 10% infill and 90% infill samples.
- In contrast, FILAFLEX FOAMY 70A, while showing slightly wear for the 30% gyroid infill density sample with an average coefficient of friction of 0.647, generally demonstrated a response of no detectable wear profile, no trace on the steel ball for all samples tested. This suggests that while some wear (material removal) might occur on the FILAFLEX FOAMY 70A surface itself (as observed for 30% infill density), significant material transfer to the counter surface is absent for both materials. The deformation characteristics of FILAFLEX FOAMY 70A indicate a greater propensity for bulk yielding or structural collapse rather than surface abrasion under these specific testing conditions.

**Figure 11:**
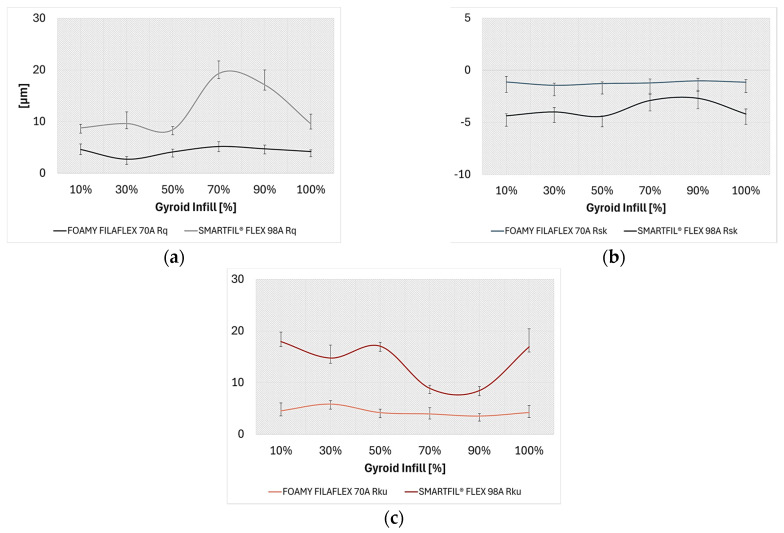
Evolution of the average amplitude parameters of TPU samples: (**a**) Average root mean square roughness (*R_q_*); (**b**) Average skewness of the profile (*R_sk_*); (**c**) Average kurtosis of the profile (*R_ku_*).

Additional roughness parameters evolution such as root mean square roughness, skewness and kurtosis, were further summarized in Figure 11a–c.

**Figure 12:**
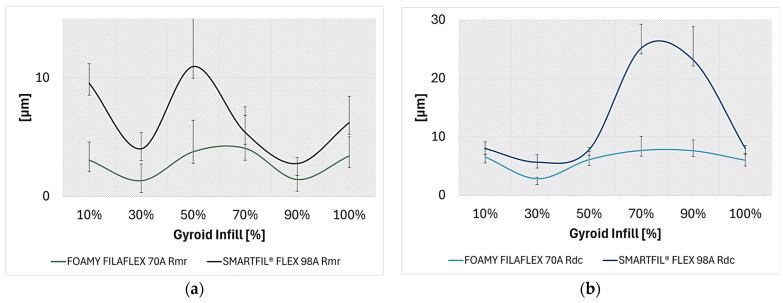
Evolution of the material ratio parameters of TPU samples: (**a**) Average material ratio (*R_mr_*); (**b**) Average profile section height difference (*R_dc_*).

These surface analysis highlight that while both TPUs resist material transfer to the steel counter surface, their underlying mechanisms of accommodating mechanical stress differ significantly. SMARTFIL^®^ FLEX 98A tends towards macroscopic deformation and collapse of its internal gyroid structures, especially at lower infill densities, resulting in a significantly rougher surface and more pronounced wear tracks. FILAFLEX FOAMY 70A, exhibiting a more stable roughness across gyroid infill densities, demonstrates surface wear for certain infill densities, while maintaining minimal adhesion or material transfer to the counter body. This suggests different performance envelopes, with Flex 98A potentially favoring applications where bulk energy absorption or deformation is desired, and FILAFLEX FOAMY 70A suitable for scenarios requiring consistent surface finish and low adhesive wear, despite local surface degradation. The evolution of the material ratio and profile height difference across TPU samples is summarized in Figure 12a,b.

### 3.5. Microscopic Characterization of TPU

Microscopic examination further revealed that FLEX 98A accommodated stress primarily through local structural collapse, whereas FOAMY 70A dissipated energy through bulk yielding and elastic recovery.

**Figure 13:**
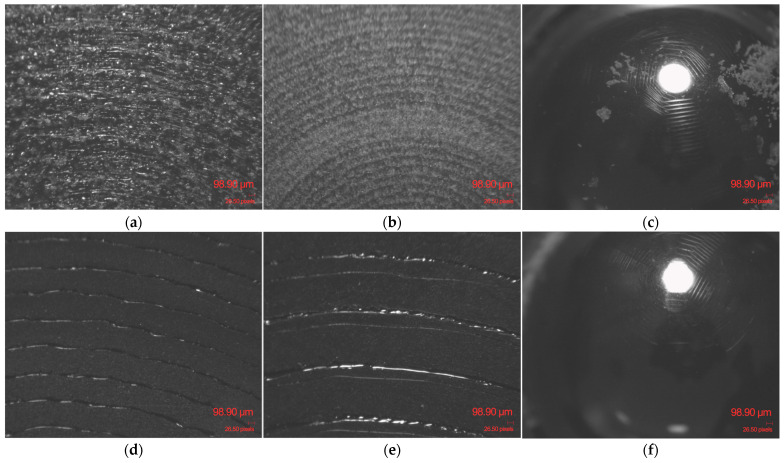
Microscopic evaluation on TPU samples and steel ball after the Ball-on-Disc test: (**a**) FOAMY FILAFLEX 70A with 10% infill specimen; (**b**) FOAMY FILAFLEX 70A with 90% infill specimen; (**c**) Counter body steel ball after tribological testing of the 90% FOAMY FILAFLEX 70A specimen; (**d**) SMARTFIL^®^ FLEX 98A with 10% infill specimen; (**e**) SMARTFIL^®^ FLEX 98A with 100% infill specimen; (**f**) Counter body steel ball after tribological testing of the 100% SMARTFIL^®^ FLEX 98A specimen.

A primary distinction lies in their different morphologies: FILAFLEX FOAMY 70A possesses an intrinsically cellular or micro-foamed structure, which results in significant internal porosity, even at high infill densities, as it can be observed in Figure 13a,b. Figure 13c illustrates the fine debris resulting from the contact interface between the steel ball and the FOAMY FILAFLEX 70A with 90% infill density specimen under the specified load and sliding distance.

The SMARTFIL^®^ FLEX 98A specimen with 10% infill density exhibits a denser and more consolidated structure, as shown in Figure 13d. By comparison, the 100% infill specimen maintains a similar structural appearance; however, following the tribological test, fine surface scratches became visible, as illustrated in Figure 13e. Correspondingly, the counter body steel ball also presented minor surface scratches after sliding against the 100% infill specimen, as depicted in Figure 13f.

This foamed nature dictates that samples at 100% infill density for FOAMY FILAFLEX 70A are expected to retain a higher proportion of voids compared to SMARTFIL^®^ FLEX 98A at 100% infill, which is likely to achieve a denser, more consolidated structure. This fundamental difference in morphology directly impacts the bulk density and, consequently, the tribological properties across the tested infill range. The consistent application of infill densities (10–100%) to both materials allows for a direct comparison of how these distinct base morphologies respond to material addition.

## 4. Discussion

### 4.1. Interpretation of Tribological Performance and Structure–Property Relationships

The tribological behavior of the two TPU materials exhibited distinct dependencies on infill density, directly reflecting their inherent material characteristics and structural responses under load.

SMARTFIL^®^ FLEX 98A demonstrated a pronounced decrease in the coefficient of friction with increasing infill density. At 10% infill, the average coefficient of friction was exceptionally high (1.174), accompanied by observable deformation of internal structures under the applied Hertzian pressure of 231 MPa. This indicates a compromise in the material’s structural integrity at low densities, leading to significant energy dissipation and higher friction as the internal gyroid lattice buckles. This aligns with the understanding that infill density directly dictates the amount of material within a 3D printed part, profoundly impacting its mechanical strength and load-bearing capacity. As infill density increased to 50%, 90% and 100%, the coefficient of friction decreased considerably, reaching values as low as 0.371 at 100% infill. This trend suggests that higher infill densities provide enhanced structural support, limiting bulk material deformation and subsequently lowering friction. Despite the significant deformation at lower infill, SMARTFIL^®^ FLEX 98A generally resisted inducing abrasive wear on the harder steel ball, with wear behavior characterized by localized plastic deformation rather than material transfer, though minor fine scratches were visible on both the 100% infill specimen and the steel ball.

By comparison, FILAFLEX FOAMY 70A, which leverages dynamic foaming technology, exhibited a consistently softer profile with a more stable yet generally higher coefficient of friction range (0.585 to 0.729) across varying infill densities. The highest average coefficient of friction for FILAFLEX FOAMY 70A was observed at 70% infill (0.729). This behavior is attributed to its intrinsic cellular or micro-foamed structure, which imparts greater surface compliance and adhesion, influencing friction differently from the denser SMARTFIL^®^ FLEX 98A. Even at 100% infill, FILAFLEX FOAMY 70A is expected to retain a higher proportion of voids compared to SMARTFIL^®^ FLEX 98A at 100% infill. Across almost all infill densities, FILAFLEX FOAMY 70A showed no detectable wear profile on the sample or mark on the steel ball, indicating minimal macroscopic wear, significant elastic recovery, or negligible material transfer. While a faint abrasion trace and slight debris were noted for the 90% infill sample, its overall response indicates a greater propensity for bulk yielding or structural collapse rather than surface abrasion under the tested conditions. This consistent behavior, despite coefficient of friction variability, underscores the unique tribological profile conferred by its foaming capability.

### 4.2. Influence of Infill Density and Surface Texture on Microscopic Features and Hardness

The surface characterization further elucidated the distinct SPP relationships of the two TPUs. SMARTFIL^®^ FLEX 98A consistently presented significantly rougher surfaces than FILAFLEX FOAMY 70A across comparable infill densities. For SMARTFIL^®^ FLEX 98A, roughness parameters such as *R_a_*, *R_q_*, *R_z_*, and *R_t_* increased substantially with increasing infill density. For instance, at 70% infill, SMARTFIL^®^ FLEX 98A showed an average *R_a_* of 10.74 µm compared to FILAFLEX FOAMY 70A’s 4.09 µm. This indicates that for SMARTFIL^®^ FLEX 98A, higher infill leads to a more pronounced topographical variation, likely due to the nature of material deposition in FFF resulting in more distinct layer lines and structural features.

Conversely, FILAFLEX FOAMY 70A maintained a relatively stable roughness magnitude across its tested infill densities (*R_a_* ranged from 1.89 µm to 4.09 µm). This stability in surface roughness is likely a direct consequence of its dynamic foaming technology, which generates a more consistent foamy texture irrespective of the internal gyroid infill density percentage.

Both TPU materials generally exhibited valley-dominated surfaces (negative skewness, *R_sk_*) and spiky profiles (kurtosis, *R_ku_* > 3), characteristic of FDM^®^ 3D printed parts due to their Archimedean pattern and inherent surface irregularities. However, SMARTFIL^®^ FLEX 98A displayed a more pronounced negative skewness (e.g., *R_sk_* −3.24 at 10% infill) and significantly higher kurtosis values (e.g., *R_ku_* 13.4 at 10% infill), indicating deeper, more prominent valleys and a much sharper distribution of peaks and valleys, especially at lower infill densities. This pronounced spikiness and valley dominance, particularly at low infill for SMARTFIL^®^ FLEX 98A, contributes to its higher initial friction and susceptibility to internal deformation.

Regarding mechanical response, the deformation of internal structures observed in SMARTFIL^®^ FLEX 98A, especially at lower infill densities, directly relates to the interplay of infill density and the material’s inherent hardness. In addition to the tribological analysis, it is important to highlight the intrinsic hardness difference between the two TPUs. SMARTFIL^®^ FLEX 98A exhibits a Shore hardness of 98A, while FILAFLEX FOAMY 70A is significantly softer, with a nominal hardness of 70A. However, due to its foaming technology, the hardness of FOAMY 70A is not fixed but can vary depending on the degree of expansion achieved during printing, ranging approximately between Shore 55A (fully foamed) and Shore 92A (unfoamed). This inherent variability explains the bulk yielding response observed in tribological tests, where FILAFLEX FOAMY 70A dissipates energy through its micro-cellular structure rather than by surface abrasion.

The foaming effect produces a cellular morphology with internal voids, leading to lower effective density and stiffness compared to the compact SMARTFIL^®^ FLEX 98A. Even at 100% infill, FOAMY 70A specimens retain a significant level of porosity, which affects their load-bearing capacity and contributes to their relatively higher and more stable friction coefficients (0.585–0.729) across infill levels. These findings underline that the distinct mechanical behavior of FOAMY 70A is closely linked to its foamed architecture, which governs its hardness, compliance, and tribological response.

Although SMARTFIL^®^ FLEX 98A is specified as a harder material (98A Shore hardness) compared to FILAFLEX FOAMY 70A (70A Shore hardness), the study highlights that without sufficient internal infill, its structural integrity is compromised, leading to bulk deformation under applied pressure. This reinforces the principle that infill density is a critical parameter for dictating mechanical strength, and its strategic use can enhance compressive strength and energy absorption. For FILAFLEX FOAMY 70A, its inherent foamed nature, which allows for stiffness adjustment between Shore 55A (fully foamed) and Shore 92A (unfoamed) through process parameters, predisposes it to a bulk yielding response rather than surface abrasion, maintaining its integrity against significant material transfer to the steel counterpart.

### 4.3. Comparative Discussion and Integration with Existing Literature

The comparative analysis reveals that while both TPUs, fabricated via FFF, exhibit characteristics common to AM (e.g., surface roughness and potential for internal porosity), their distinct material compositions and responses under tribological loading lead to divergent performance profiles. The general understanding that AM enables the fabrication of complex geometries from digital models through a layer-by-layer approach is further refined by this study, which demonstrates how such architectural control facilitates the functional customization of components.

The SMARTFIL^®^ FLEX 98A, with its denser structure at higher infill densities, exemplifies a material suitable for robust, deformable components where high structural integrity and controlled deformation under stress are paramount. Its sharp decrease in coefficient of friction with increasing infill aligns with Norani’s [11] observation that infill densities correlate with increased strength and viscosity due to improved adhesive bonding between polymer molecules. This capability is mandatory for engineering applications requiring resilience, such as those subject to significant stress or vibration, where excellent inter-layer adhesion and high impact resistance are desired.

Conversely, FILAFLEX FOAMY 70A, with its intrinsic foaming technology and consistently stable surface roughness across infill densities, is better suited for scenarios demanding a consistent surface finish and low adhesive wear. This behavior highlights the potential of advanced foaming technologies to manipulate 3D print properties, allowing for material stiffness control by adjusting process parameters. The consistent low wear on the steel counterpart observed for FILAFLEX FOAMY 70A supports its application in environments where material transfer is undesirable. The ability to vary stiffness systematically through infill density and patterns, as demonstrated for these TPUs, is particularly instrumental in producing functionally graded materials (FGMs), with practical utility in developing personalized orthoses and wheelchair cushions.

The study addresses a noted gap in the literature regarding the comprehensive understanding and optimization of SPP relationships in TPU components, particularly the limited research on the tribological properties of 3D printed polymers. By integrating the influence of gyroid infill density and Archimedean surface texture on two distinct TPU types, this work provides comprehensive data that advances the design and manufacturing of customized, high-performance polymeric components for diverse applications where variable stiffness and controlled friction are important. The observed mechanisms of internal structural deformation and bulk yielding, rather than significant abrasive wear on the counter-body, offer novel insights into the specific tribological responses of flexible polymers under contact, which differs from conventional wear mechanisms often studied in harder materials.

### 4.4. Scientific Significance and Future Research Directions

This investigation significantly contributes to the field of polymer science and additive manufacturing by providing a detailed, comparative analysis of two commercially relevant TPUs under tribological loading. The scientific significance lies in elucidating how internal architecture (infill density and pattern) and inherent material characteristics (foamed vs. solid, different hardness) synergistically dictate tribological performance and surface morphology. This directly informs the development of sturdier predictive models and optimized printing strategies for flexible polymeric materials, facilitating their broader industrial adoption.

The findings have considerable implications for applications, particularly in the design of functionally graded materials (FGMs) where specific mechanical and tribological profiles are required within a single component. The distinct application profiles identified for SMARTFIL^®^ FLEX 98A (robust, deformable components) and FILAFLEX FOAMY 70A (consistent surface finish, low adhesive wear) offer clear guidance for engineers and designers. For instance, applications requiring impact absorption or flexible interfaces could benefit from SMARTFIL^®^ FLEX 98A with optimized infill, while soft contact applications demanding consistent friction and minimal debris might favor FILAFLEX FOAMY 70A.

Future research directions should include a more in-depth analysis of the visco-elastic contributions to the observed friction and deformation mechanisms, particularly for the foamed TPU. Further quantitative correlation between specific foaming agent activation parameters (e.g., printing temperature, speed, flow ratio) and the resulting micro-cellular structure’s impact on tribological properties would enhance the precision of material design. Also, exploring the long-term wear behavior and fatigue resistance of these materials under cyclic loading conditions would provide a more complete picture of their durability in demanding applications. Investigating the influence of other advanced infill patterns and surface textures, along with varying environmental conditions, could further broaden the understanding of SPP relationships in 3D printed TPUs.

The pronounced decrease in coefficient of friction with increasing infill density observed for SMARTFIL^®^ FLEX 98A is consistent with findings by [11], who reported that higher infill ratios improve molecular bonding and reduce bulk deformation under load. Conversely, the stable yet generally higher coefficient of friction values of FILAFLEX FOAMY 70A differ from previous studies on solid TPUs [12], suggesting that its cellular architecture introduces additional compliance and adhesive interactions. The negative skewness values (*R_sk_* < 0) measured for both TPUs indicate valley-dominated surfaces, which may aid in debris entrapment and partial load distribution, while the high kurtosis (*R_ku_* > 3) reflects spiky profiles typical of FDM^®^-produced surfaces.

Despite the robustness of these findings, the study has several limitations. Only two TPU types were investigated, under dry sliding conditions and limited to short-duration tests. Long-term fatigue and cyclic wear behaviour remain unexplored. Furthermore, the influence of alternative infill geometries (e.g., honeycomb, cubic) and surface post-processing techniques was not assessed. Future work should address these aspects, alongside testing under lubricated conditions and extending the investigation to biomedical and protective equipment applications where TPU components are subject to repetitive loading and variable environments.

## 5. Conclusions

In summary, this study demonstrates that tribological behavior in 3D-printed TPU is dictated by both material morphology and infill density. SMARTFIL^®^ FLEX 98A, with its higher hardness and denser architecture, is suitable for energy-absorbing and deformable components requiring structural integrity, while FILAFLEX FOAMY 70A offers stable surface performance and minimal adhesive wear, even at high infill. These findings provide practical guidelines for designing functionally graded TPU structures in applications ranging from automotive damping elements to customized orthoses and protective sports equipment.

This study demonstrated that the tribological and surface behavior of TPUs strongly depends on both material type and infill density. SMARTFIL^®^ FLEX 98A showed a marked decrease in friction coefficient with increasing infill, reflecting its higher load-bearing capacity and reduced structural deformation at high densities. In contrast, FILAFLEX FOAMY 70A maintained more stable coefficient of friction values, influenced by its foamed morphology and bulk yielding tendency. Surface analysis confirmed that SMARTFIL^®^ FLEX 98A develops rougher surfaces with increasing infill, while FILAFLEX FOAMY 70A retains relatively constant roughness. Both materials resisted abrasive wear on the steel counterpart, but accommodated stress through different mechanisms. These findings suggest distinct application niches: SMARTFIL^®^ FLEX 98A is recommended for robust, deformable structures requiring impact absorption, whereas FILAFLEX FOAMY 70A is better suited for components where consistent surface finish and low adhesive wear are critical. Overall, the study advances the optimization of functionally graded TPU components by linking infill architecture and surface features to performance outcomes.

## Figures and Tables

**Table 1:** Thermoplastic Polyurethane (TPU) Filaments Comparative Physical and Mechanical Properties According to Manufacturer Data Sheets.

Material Property	SMARTFIL^®^ FLEX 98A	FILAFLEX FOAMY 70A	References
Density	1.09 g/cm^3^	1.05 g/cm^3^	[32,33]
Tensile Strength	XY plane: 16.8 MPa ZX plane: 6.8 MPa	37 MPa	[32,33]
Tensile Modulus	XY plane: 4.7 MPa ZX plane: 9.1 MPa	N/A *	[32]
Flexural Strength	XY plane: 8 MPa ZX plane: 5.1 MPa	N/A	[32]
Flexural Modulus	XY plane: 50.1 MPa ZX plane: 60.2 MPa	N/A	[32]
Elongation at Break	XY plane: 244.5% ZX plane: 71.4%	740%	[32,33]
Flexural Strain	XY plane: 15.4% ZX plane: 15.4%	N/A	[32]

* N/A Value not available on manufacturer data sheet.

**Table 2:** Thermoplastic Polyurethane Specimens’ Printing Parameters.

Printing Parameters	SMARTFIL^®^ FLEX 98A	FILAFLEX FOAMY 70A
Nozzle Temperature	230 °C	230 °C
Build Plate Temperature	Off	Off
Extrusion/Printing Speed	30 mm/s	20 mm/s

**Table 3:** Summary of Tribological Results for FILAFLEX FOAMY 70A Obtained via Ball-on-Disc Testing.

Gyroid Infill Density [%]	$\bar{\mu }$ * ± σ **	$\mu_{min}$	$\mu_{max}$
10	0.59 ± 0.10	0.48	0.90
30	0.65 ± 0.05	0.61	0.84
50	0.65 ± 0.04	0.59	0.78
70	0.73 ± 0.07	0.48	0.79
90	0.72 ± 0.07	0.48	0.76
100	0.65 ± 0.06	0.45	0.69

* $\bar{\mu }$ refers to the average coefficient of friction. ** *σ* refers to standard deviation.

**Table 4:** Summary of Tribological Results for SMARTFIL^®^ FLEX 98A Obtained via Ball-on-Disc Testing.

Gyroid Infill Density [%]	$\bar{\mu }$ * ± σ **	$\mu_{min}$	$\mu_{max}$
10	1.17 ± 0.24	0.47	1.45
30	0.57 ± 0.12	0.26	0.74
50	0.38 ± 0.09	0.37	0.40
70	0.52 ± 0.04	0.41	0.57
90	0.40 ± 0.03	0.29	0.41
100	0.37 ± 0.03	0.36	0.49

* $\bar{\mu }$ refers to the average coefficient of friction. ** *σ* refers to standard deviation.

**Table 5:** Average Amplitude Parameters and Roughness Profile of FILAFLEX FOAMY 70A Specimens at Different Gyroid Infill Densities.

Gyroid Infill [%]	$\bar{R_{p}}$ * ± *σ* ** [µm]	$\bar{R_{v}}$ * ± *σ* [µm]	$\bar{R_{z}}$* ± *σ* [µm]	$\bar{R_{c}}$ * ± *σ* [µm]	$\bar{R_{t}}$ * ± *σ* [µm]	$\bar{R_{a}}$ * ± *σ* [µm]	$\bar{R_{q}}$ * ± *σ* [µm]	$\bar{R_{sk}}$ * ± *σ*	$\bar{R_{ku}}$ * ± *σ*
10	5.9 ± 1.21	13.33 ± 2.00	19.23 ± 2.95	11.1 ± 1.74	24.97 ± 3.27	3.6 ± 0.98	4.6 ± 1.07	−1.13 ± 0.54	4.55 ± 1.52
30	4.07 ± 1.02	9.72 ± 1.64	13.79 ± 2.65	6.90 ± 1.29	19.30 ± 6.80	1.89 ± 0.33	2.71 ± 0.52	−1.45 ± 0.21	5.85 ± 0.65
50	5.38 ± 0.63	12.69 ±1.40	18.06 ± 1.88	12.1 ±1.73	22.13 ± 2.36	3.21 ± 0.55	4.13 ± 0.53	−1.28 ± 0.16	4.20 ± 0.64
70	6.38 ± 1.94	14.73 ± 1.52	21.13 ± 2.72	13.9 ± 1.25	27.26 ± 3.76	4.09 ± 0.93	5.18 ± 1.01	−1.22 ± 0.38	3.94 ± 1.20
90	6.47 ± 0.99	12.86 ± 2.05	19.33 ± 2.56	12.55 ± 2.95	24.40 ± 0.09	3.78 ± 0.71	4.73 ± 0.81	−1.02 ± 0.24	3.53 ± 0.45
100	5.37 ± 0.69	12.90 ± 1.35	18.26 ± 1.61	9.95 ± 1.50	24.53 ± 3.35	3.24 ± 0.33	4.20 ± 0.16	−1.15 ± 0.26	4.22 ± 1.37

* Refers to average measured values. ** *σ* refers to standard deviation.

**Table 6:** Material Ratio Average of FILAFLEX FOAMY 70A Specimens at Different Infill Densities.

Gyroid Infill [%]	$\bar{R_{mr}}$ * ± *σ* ** [%]	$\bar{R_{dc}}$ * ± *σ* [µm]
10	3.07 ± 1.51	6.55 ± 2.57
30	1.33 ± 1.40	2.83 ± 0.27
50	3.77 ± 2.66	6.10 ± 1.38
70	4.06 ± 3.50	7.67 ± 2.42
90	1.43 ± 0.35	7.61 ± 1.84
100	3.42 ± 1.61	5.99 ± 1.13

* Refers to average measured values. ** *σ* refers to standard deviation.

**Table 7:** Average Amplitude Parameters and Roughness Profile of SMARTFIL^®^ FLEX 98A Specimens at Different Gyroid Infill Densities.

Gyroid Infill [%]	$\bar{R_{p}}$ * ± *σ* ** [µm]	$\bar{R_{v}}$ * ± *σ* [µm]	$\bar{R_{z}}$* ± *σ* [µm]	$\bar{R_{c}}$ * ± *σ* [µm]	$\bar{R_{t}}$ * ± *σ* [µm]	$\bar{R_{a}}$ * ± *σ* [µm]	$\bar{R_{q}}$ * ± *σ* [µm]	$\bar{R_{sk}}$ * ± *σ*	$\bar{R_{ku}}$ * ± *σ*
10	3.01 ± 0.28	19.50 ± 1.96	22.53 ± 2.21	17.03 ± 4.82	24.63 ± 1.66	2.34 ± 0.48	4.17 ± 0.71	−3.24 ± 0.20	13.4 ± 1.83
30	5.13 ± 1.59	25.93 ± 6.43	31.07 ± 8.03	27.83 ± 8.61	37.03 ± 4.68	4.52 ± 1.81	6.90 ± 2.26	−2.55 ± 0.43	8.91 ± 2.50
50	3.21 ± 0.47	19.86 ± 2.48	23.06 ± 3.00	15.83 ± 5.00	25.23 ± 2.30	2.41 ± 0.39	4.30 ± 0.61	−3.14 ± 0.05	12.86 ± 0.73
70	12.89 ± 2.83	46.10 ± 5.24	59.00 ± 7.99	45.40 ± 14.6	65.57 ± 7.53	10.74 ± 2.0	14.13 ± 2.43	−1.68 ± 013	4.94 ± 0.59
90	11.14 ± 2.32	38.63 ± 6.31	49.83 ± 8.46	37.1 ± 10.21	57.23 ± 10.21	9.43 ± 2.5	12.37 ± 2.91	−1.67 ± 0.17	4.93 ± 0.77
100	4.04 ± 1.21	24.20 ± 5.21	28.23 ± 6.31	23.63 ± 9.65	35.40 ± 6.67	3.16 ± 1.51	5.34 ± 1.91	−3.05 ± 0.49	12.71 ± 3.49

* Refers to average measured values. ** *σ* refers to standard deviation.

**Table 8:** Material Ratio Average of SMARTFIL^®^ FLEX 98A Specimens at Different Infill Densities.

Gyroid Infill [%]	$\bar{R_{mr}}$ * ± *σ* ** [%]	$\bar{R_{dc}}$ * ± *σ* [µm]
10	6.47 ± 1.65	1.43 ± 0.09
30	2.69 ± 1.36	2.81 ± 1.29
50	7.19 ± 4.64	1.72 ± 0.38
70	1.34 ± 1.43	17.5 ± 4.10
90	1.34 ± 0.50	15.5 ± 5.71
100	2.80 ± 2.22	2.00 ± 0.55

* Refers to average measured values. ** *σ* refers to standard deviation.

## Data Availability

The original contributions presented in this study are included in the article. Further inquiries can be directed to the corresponding author.

## Abbreviations

The following abbreviations are used in this manuscript:

AM	Additive Manufacturing
ANOVA	Analysis of Variance
ASTM	American Society for Testing and Materials
CAD	Computer-Aided Design
DC	Digital Camera
DIN	Deutsches Institut für Normung (German Institute for Standardization)
FDM^®^	Fused Deposition Modeling
FFF	Fused Filament Fabrication
FGM	Functionally Graded Material
HS	Hard Segments
ISO	International Organization for Standardization
MEX	Material Extrusion
RH	Relative Humidity
*R_a_*	Arithmetic Mean Roughness
*R_c_*	Average Mean Height of Profile Elements
*R_dc_*	Reduced depth of the roughness profile
*R_q_*	Root Mean Square Roughness
*R_mr_*	Material Ratio
*R_t_*	Total Height of Profile
*R_p_*	Maximum Profile Peak Height
*R_v_*	Maximum Profile Valley Depth
*R_sk_*	Roughness Skewness
*R_ku_*	Roughness Kurtosis
*R_z_*	Maximum height of profile
SPP	Structure–Property–Performance
SS	Soft Segments
STL	Stereolithography File Format
TPU	Thermoplastic Polyurethane
